# Pandemic management: Analysis of availability and relevance of surveillance indicators by COVID-Task-Forces in the German federal state of Lower Saxony

**DOI:** 10.1016/j.infpip.2023.100294

**Published:** 2023-06-15

**Authors:** Nicolás Reinoso Schiller, Karina Usipbekova, Katja Hille, Johannes Dreesman, Kjell Schwarz, Karin Reimers, Fabian Feil, Simone Scheithauer

**Affiliations:** a Department of Infection Control and Infectious Diseases, University Medical Center Göttingen, Göttingen, Germany; b Public Health Agency of Lower Saxony (NLGA), Hanover, Germany

**Keywords:** COVID-19, Organizational decision making, Sentinel surveillance, Public health service, Cognitive bias

## Abstract

**Background:**

Locally, the introduction of measures during times of a pandemic emergency is embodied in a pandemic containment plan created by the Robert Koch Institute in 2017. In addition to central indicators such as incidence rates and number of deaths, various indicators are used at the local level to assess the pandemic situation. So far, there hasn't been analyses of the availability and perceived relevance of the surveillance indicators used to manage the SARS-CoV-2 pandemic by the local German pandemic task forces.

**Aim:**

This study examined whether local decision-makers had access to surveillance-related indicators in a way that they could be used to make informed decisions in response to the pandemic situation.

**Methods:**

A cross sectional study was conducted, using an online questionnaire developed by experts of The Public Health Agency of Lower Saxony and The University Medical Center Göttingen (UMG). All local COVID-19 task forces of the German state of Lower-Saxony were enrolled in the study.

**Findings:**

The surveillance indicators assessed by survey respondents as most available and relevant are included under the German Infection Protection Act (IfSG). In contrast, the indicators that are not bound by the IfSG have a significantly lower availability and an inconsistent assessment of relevance.

**Conclusion:**

Against the background of efficiency, it seems central to be able to reliably provide the highly weighted surveillance indicators. Nevertheless, the relevance assessment gap between the indicators embedded in the IfSG and the ones that are not may be explained by cognitive processes such as anchoring bias. The collection and use of indicators to assess the pandemic situation and to evaluate measures should be the subject of continuous multidisciplinary discussions.

## Introduction

In March 2020, the World Health Organization (WHO) declared the severe acute respiratory syndrome coronavirus type 2 (SARS-CoV-2) disease as the biggest pandemic of the 21st century as it rapidly spread around Europe and the world, causing the biggest health crisis in Europe since the Second World War [1,2]. Quickly, measures to mitigate the spread of the virus were taken all around the globe with different degrees of severity of restrictions for the population and in accordance to risk assessments from the WHO, the European Center for Disease Control and Prevention (ECDC), the US Center of Disease Control and Prevention (CDC) and other major health institutions [3].

In Germany, the protection against infection act (Infektions schutzgesetz, IfSG) provides the legal basis for infection prevention and control measures [4]. This law reflects the hierarchical organization of the German Public Health sector where three administrative levels are involved. Authorities at the level of administrative districts and independent cities have the main responsibilities for carrying out infection protection measures. This applies particularly to measures, for which direct contact or communication with the citizens affected by the disease is necessary, because these local authorities receive the notification data and only they have the cases' personal identification data [5]. The federal states have strong responsibilities in organizing and financing the supply of medical services, but have little responsibilities according to the IfSG in routine infection prevention. The national level defines the rules and standards, e.g. of the notification system, and formulates recommendations for hygiene, vaccination provision etc. [6].

In the event of a pandemic, additional tasks have to be organized, like procurement, distribution and application of vaccines, provision of test possibilities, and protection of critical infrastructure. For this purpose, specific management plans were developed, mainly to be prepared for influenza pandemics. The process of developing such plans is guided by the recommendations given by the WHO [7]. The latest pre-pandemic version of the German pandemic preparedness plan was developed in cooperation of the federal state health ministries and the national health ministry and published in 2017 [8]. After the start of the SARS-CoV-2- pandemic, a specific supplementary plan for COVID-19 was developed and published by the national public health institute Robert Koch-Institut [9].

According to the German pandemic plans, the responsibility for dealing with cases, but also the organization of local vaccination centers, of rescue services, of public transport or the protection of critical infrastructure are the responsibility of the administrative districts and independent cities [10]. For this purpose, the local administrations have organized themselves by establishing task forces, ensuring the rapid and adequate response to the challenges during the pandemic [11]. These specially created COVID-19 Task Forces (CTFs) were thus organized district wise and included members of different professional backgrounds.

However, even with a carefully structured crisis plan, created to be carried out in a high-income country with well-prepared professionals and a strong health care system such as the German public health system, the pandemic situation exceeded the possibilities of many health centers and hospitals [12,13]. In order to better understand whether relevant indicators are available for the decision-making processes of CTFs or whether information gaps exist, an analysis of the available indicators used in the management of the COVID-19 pandemic is essential.

The main objective of the study was to identify which surveillance-related indicators were available to decision makers of local districts and independent cities in the German state of Lower Saxony and whether or not those indicators could be considered usable (as opposed to “unusable due to not being available in time to be included in a given assessment”). In addition, the study explored the perceived relevance of the proposed surveillance indicators, some of which are partially missing and could be of substantial value for the decision-making process of local CTFs.

## Methods

### Study design

The current article has been developed as part of the “Federal Research Network for Applied Surveillance and Testing” (B-FAST, with grant number: 01KX2021) and PREparedness and PAndemic REsponse in Deutschland (PREPARED, with grant number: 01KX2121) from the German Network of University Hospitals (NUM) in order to elucidate which indicators were considered relevant and available in a timely manner for decision makers. For this purpose an explorative, cross-sectional observational study involving all CTFs in the German federal state of Lower Saxony was conducted. Participation was anonymous. The project was approved by the local committee under the file no. 5/2/21 with a positive ethical consent.

### Recruitment and study population

The online survey was distributed by the Public Health Agency of Lower Saxony (NLGA^3^) and the Lower Saxony County Council with a timeframe for return between August and September of 2021 to all 45 Lower Saxony CTFs.

### Survey

The survey was developed by a team of experts in epidemiology, public health, infection control, infectious diseases, hygiene and psychology from the Department of Infection Control and Infectious Diseases at the University Medical Center Göttingen (UMG^4^) and the NLGA. The survey was conducted using the online survey tool LimeSurvey (https://www.limesurvey.org/).

All study participants were asked to answer whether or not the CTF of which they are a member, received information about certain indicators. They were also asked to assess the relevance of these indicators or whether these indicators are relevant, but cannot be used due to e.g. late arrival of the information to the CTF.

The questionnaire consisted of six main parts, 1) a general part about the district and the CTF organization, parts 2–5 about indicators on 2) SARS-CoV-2 testing, 3) hospital capacity, 4) cases in care facilities, 5) vaccination, and part 6) on additional information and interventions.

### Data and statistical analysis

For the statistical analysis the program “R studio” in its version 1.4.1717 for macOS was used.

Since the number of responses varied within the questions on availability and relevance, ratings were created and reported as fractions, displaying the total of affirmative answers received (numerator) and the total of received answers for the specific indicator (denominator). These ratings were used to divide the indicators into two groups, one group with high indicator availability ratings and the other with low indicator availability.

Given that the data was not distributed according to a Gaussian distribution, the non-parametric test “Mann-Whitney-U-Test” was chosen to calculate the differences between the two groups explained above.

## Results

A total of 35 of the targeted 45 CTFs within the districts and independent municipalities of Lower Saxony responded to the survey, thus reflecting a response rate of 78%.

**Figure 1:**
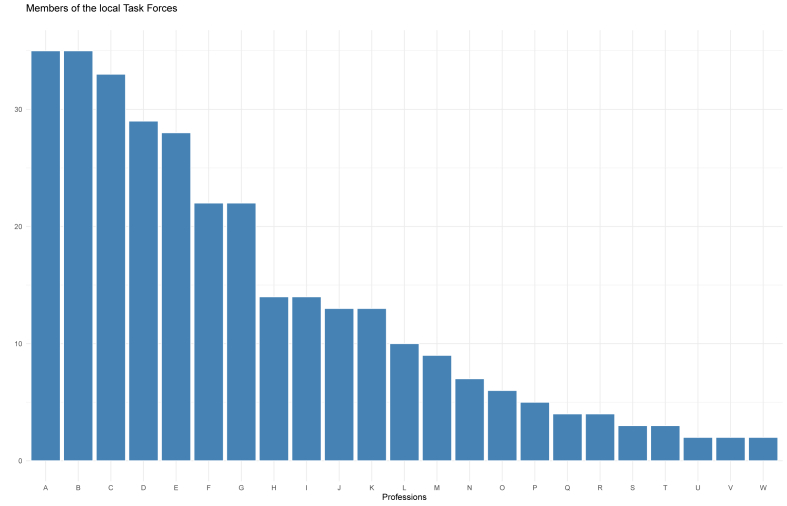
Bar plot displaying the professions included on the CTFs that participated in the survey.

The CTFs that participated in the survey were made up of the following professionals and organizations: A) Health Department (35 CTFs), B) Civil Protection (35), C) Public Order Office (33), D) District Administrator (29), E) Police (28), F) Aid Organizations (22), G) Fire Brigade (22), H) School Board (14), I) Hospitals (14), J) Press Office (13), K) Care Home Supervisor (13), L) Technical Relief Agency (10), M) Federal Armed Forces (9), N) Veterinary Office (7), O) Rescue Service (6), P) Staff Councils or Personnel Office (5), Q) Youth Welfare Office (4), R) IT Service (3), as well as single representatives of the S) business community, T) Non-governmental organizations, U) Laboratory Center, V) Association of Statutory Health Insurance Physicians and W) Physicians in private practices (see Fig. 1).

The current analysis will focus on the indicators related to surveillance in hospitals, surveillance in nursing homes and care for the elderly. A list of all indicators can be found in the appendix.

### Indicators regarding the surveillance in hospitals

Due to the amount of data gathered with the survey, we will report the most relevant indicators in this section, i.e. we will focus first on the gaps on availability and not on the existing indicators that are available and can be used in all CTFs.

There was no indicator which was stated as not available by all CTFs, however the indicator “**Number of people who are treated daily in the emergency department” was** the least available indicator. Only 5 of the 29 total answers reported the indicator as available while the reported relevance was 9/29. In addition, two of the five CTFs that claimed to have data for this parameter could not use them in a timely manner. A similar situation was observed for the indicator **“Number of people coming to the emergency department every day with suspected SARS-CoV-2 infection”**. Only 8 participants reported having this data (8/28), of which two were unable to make use of it in a timely manner. The number of statements classifying this indicator as relevant was 19 out of 29 answers (19/29). The indicator **“Total number of new patients admitted daily (independent of COVID-19)”**, with a relevance rating of 13/31 is available in 10 CTFs (10/29), 3 of which didn't receive the data in time.

**Figure 2:**
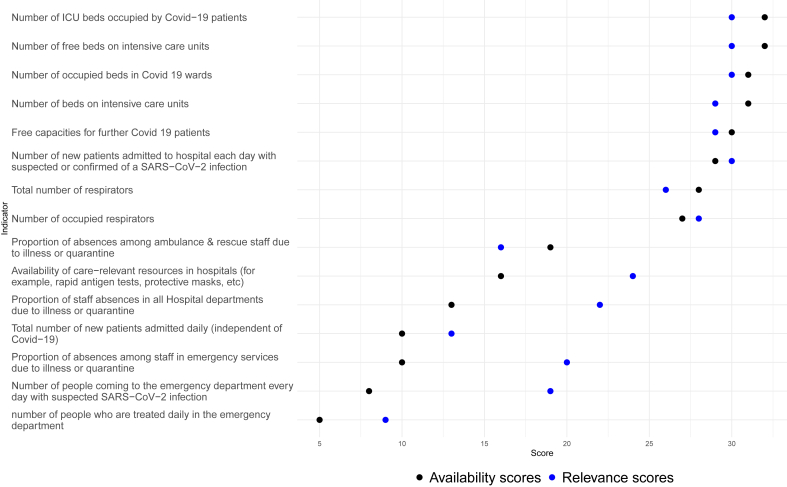
Absolute count of affirmative answers on availability and relevance for each indicator related to hospital surveillance.

For the indicator **“Proportion of absences among staff in emergency services due to illness or quarantine”**, 10 CTFs stated that they did have the data for this indicator (10/29) while one CTF stated that they had the data but could not make use of it in a timely manner. This indicator was reported 20 times as relevant of a total of 29 relevance statements (20/29). A similar rating of data availability vs. total answers can be found for the indicators: **“Proportion of hospital staff absences due to illness or quarantine”** (13/31) and a relevance rating of 22/30, while **“Availability of care-relevant resources in hospitals (for example, rapid antigen tests, protective masks, etc)”** had a data availability rating of 16/30 and relevance rating of 24/30 (see Figure 2).

Turning now to the indicators with high availability, the most widely available indicator was the **“Number of ICU beds occupied by COVID-19 patients”** (32/33) with a relevance rating of 30/33, did not get any statement on the delay of the information and only one participating CTFs stated that they did not have this data.

The indicators **“Number of occupied beds in COVID 19 wards”** (availability: 31/33) with relevance rating 30/33, **“Number of free beds on intensive care units”** (availability: 32/33) with a relevance of 30/33 and **“Number of beds on intensive care units”** (availability: 31/33) with a relevance of 29/33, were also among the most available indicators. The availability and relevance of all surveillance indicators included in the survey can be seen in Figure 2.

The hospital surveillance indicators can be divided into two groups: The first group encompasses all indicators where the majority of statements display a higher score on availability vs. relevance. The second group consists of all indicators with a higher relevance rating and a lower availability statement (see Figure 2, black vs. blue). The availability gap between both groups (*P* < 0.05) as well as the relevance gap (*P* < 0.05) was tested significant with the non-parametric Mann-Whitney U test, suggesting that the differences in availability and relevance between both groups are not random but rather systematic.

### Indicators for the surveillance in nursing homes and care for the elderly

For the nursing home and elder care monitoring indicators, the CTFs reported high levels of availability. For both indicators with the highest availability (32/34) “**Number of daily newly confirmed cases of SARS-CoV-2 infection among residents of inpatient geriatric and nursing facilities**” (relevance: 30/34) and “**Number of daily newly confirmed cases of SARS-CoV-2 infections among employees of inpatient care and nursing facilities**” (relevance: 29/34), it was reported a lack of the indicator (2 CTFs for each indicator) and “non-timely availability” of the indicator (also two CTFs each).

**Figure 4:**
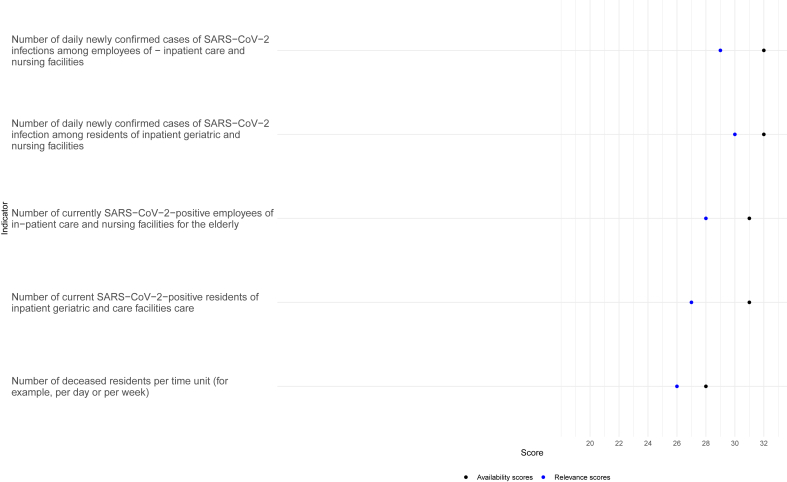
Absolute counts for the relevance and availability of each of the surveillance indicators on the “nursing homes and care for the elderly” category.

The indicator “**Number of current SARS-CoV-2-positive residents of inpatient geriatric and care facilities care**” with a rated relevance of 27/34 and an availability rating of 31/34 was reported as a missing indicator by 3 CTFs. **“Number of currently SARS-CoV-2-positive employees of in-patient care and nursing facilities for the elderly”** was declared relevant by 28 of 34 experts and it was mentioned as available 31 times, unavailable 3 times (31/34) and available but not in a timely manner 3 times. The least available indicator within this category was the **“Number of deceased residents per time unit (for example, per day or per week)”** with an availability rating of 28/33 with two CTFs reporting non-availability on a timely manner and a relevance rating of 26/33 (see Figure 4).

## Discussion

Overall, the high level of consistency between the ratings of availability and relevance of the indicators illustrates a good level of exchange between the various stakeholders who record and transmit data (test and vaccination centers, hospitals, care facilities) and the CTFs.

**Figure 3:**
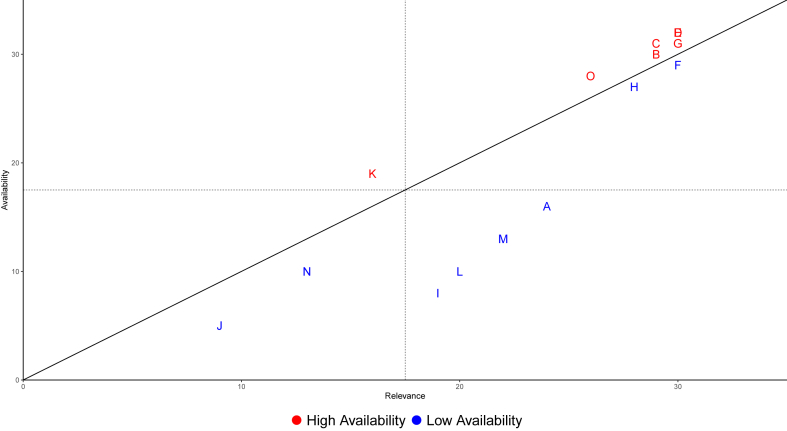
Relationship between availability and relevance of indicators in absolute counts of affirmative answers. (A) Availability of care-relevant resources in hospitals (for example, rapid antigen tests, protective masks, etc). (B) Free capacities for further COVID-19 patients. (C) Number of beds on intensive care units. (D) Number of free beds on intensive care units. (E) Number of ICU beds occupied by COVID-19 patients. (F) Number of new patients admitted to hospital each day with suspected or confirmed SARS-CoV-2 infection. (G) Number of occupied beds in COVID19 wards. (H) Number of occupied respirators. (I) Number of people coming to the emergency department every day with suspected SARS-CoV-2 infection. (J) Number of people who are treated daily in the emergency department. (K) Proportion of absences among ambulance & rescue staff due to illness or quarantine. (L) Proportion of absences among staff in emergency services due to illness or quarantine. (M) Proportion of staff absences in all Hospital departments due to illness or quarantine. (N) Total number of new patients admitted daily (independent of COVID-19). (O) Total number of respirators.

The systematic difference between the low availability and high availability groups (Figure 3) could be explained by the fact that the indicators with high availability are mostly reported mandatory according to the German Infection Protection Act (IfSG). The only two indicators that are not mandatory reported at the time of the survey but nevertheless have a high rating for availability as well as for relevance are “Number of occupied respirators” and “Number of new patients admitted to hospital each day with suspected or confirmed SARS-CoV-2 infection” (see Figure 3).

Nearly all ICU and “Hospital COVID-19 capacity” reported data were given on time; these findings could be explained by three main aspects. The first aspect is the mandatory electronic reporting of such data established by the central government and managed by the RKI, that obligate all hospitals to publish their data in central databases such as the intensive care database “DIVI register”.^5^ The indicator “Number of free beds on intensive care units” is the only one indicator related to the ICUs with a mention of delayed provision of data, that due to the easy accessibility of this data on the DIVI register, can be interpreted as lack of knowledge by the respondent rather than an actual delay on the availability of the data. Further reasons could be insufficient digitization standards of the German health services and a poor digital literacy of the personnel in hospitals and public services [11,14].

From a multidisciplinary point of view, the statistical difference between the groups with high and low availability (Figure 3) on their relevance ratings could be the result of an anchoring bias, a predisposition to over rely on available information [15]. The anchoring bias could explain why known and available indicators are used (and rated as relevant) while unavailable (or unknown) indicators might be rated as not relevant. Cognitive biases, groupthink, and misperceptions, as well as political reaction, can conflict with logical and impartial risk assessment. These issues have already been discussed with regard to general policy decision situations [16], but also in the context of the SARS-CoV-2 pandemic [10,17]. Further work is needed to determine the extent of the impact of the anchoring bias and other potential cognitive biases on the perceived relevance of the surveillance indicators.

In the results presented here, anchoring bias could have an impact on indicators such as “Proportion of staff absences in all hospital departments due to illness or quarantine”, “Proportion of absences among staff in emergency services due to illness or quarantine” and “Availability of care-relevant resources in hospitals (for example, rapid antigen tests, protective masks, etc)”. Although the three indicators have had the highest reported relevance among the category of “low availability” indicators there is almost ten points of difference between the average relevance to the “high availability” indicators and their relevance statements, meaning that almost one third of the participants did not consider these factors to be relevant for managing the SARS-CoV-2 pandemic. However, they can be critical for the assessment of work overload [18] or for mental health and stress problems of the staff [19]. Furthermore, “Availability of care-relevant resources in hospitals (for example, rapid antigen tests, protective masks, etc)” has been reported as a key indicator to the work of all CTFs by the RKI and the German federal government [10,20] with a low reported availability (16/30) and should therefore be further addressed by stakeholders and future research. For the indicators “Total number of new patients admitted daily (independent of COVID-19)” and “Number of people who are treated daily in the emergency department” it could be argued that they are extremely helpful as predictive indicators in simulation models assessing the management of the pandemic (or epidemic) situation and can have a harmful impact on hospital mortality [[21], [22], [23]]. Therefore their significantly lower relevance ratings could be in fact a consequence of an anchoring bias. Furthermore, the least available indicator and with the lowest relevance rating “Number of people who are treated daily in the emergency department” seems to be significantly influenced by respiratory viral epidemics and mediated by seasonal variations [24], however, it may confound with other indicators which were not part of this study such as the ARI (acute Respiratory Infections) surveillance indicators.

Now focusing on the indicators relevant to the surveillance in nursing homes and care for the elderly, we find a completely different situation: all indicators that have been examined were widely available. This could be due to the fact that all assessed indicators were reported mandatory according to the German Infection Protection Act and therefore, all stationary care and nursing facilities had the obligation to report them. However, despite the wide availability of the indicators, all of them have had at least two reports of data not being timely available. This fact could be explained by the same two aspects as for the hospital surveillance: the low digitization standards of the German health services and the insufficient digital literacy by the personnel in hospitals, care facilities and public services [11,14].

Although the indicators for surveillance in nursing homes and care for the elderly were found to be relevant and had a high correlation between availability and perceived relevance, there may be an anchoring bias for the indicator “Number of deceased residents per time unit (for example, per day or per week)”, as the indicator was almost exclusively rated as relevant by the same group of CTFs that reported its availability.

## Limitations

Cross-sectional studies have a number of inherent limitations, which can also be found in this research. The first limitation typically found in such a study design is that results depend largely on the time where the survey was conducted and the given information flow [25] to the participants at that time. In the specific case of this study, the professional backgrounds of the participants could play a role in the knowledge that they possess over certain questions or indicators. Also, responses could be affected by recall and biases, which implies a poor recall of the information held by the respondents. Even though the response rate was high (75%), selection bias may influence the result as not all CFTs in Lower Saxony have answered the survey. The indicators presented by the researchers for their evaluation were limited in number and do not explore the totality of the possibly relevant surveillance indicators.

The generalisability of the data is limited, partly due to the fact that only CTFs from Lower Saxony were addressed and each federal state has had different measures and approaches during the pandemic.

## Conclusions

To conclude, there is a good exchange between the various stakeholders who record and transmit data and the CTFs. However, our findings suggest that cognitive biases, group thinking and misperceptions can play an important role in regional pandemic management [10] and should be studied in greater detail for local and regional policymakers. In addition, the lack of digital expertise and infrastructure as well as personal capacities of the hospital, nursing homes and public agencies staff and the already known interoperability and digitalization obstacles of data integration between hospitals and German public agencies [14] could quickly become marginal with specific central policies. A continuous process of evaluation of the indicators used to manage the pandemic situation would also lead to an enhanced data quality and integration which might be beneficial for secondary data use, e.g. scientific analyses and epidemiological assessments [26]. In addition, a continuous evaluation of specific indicators could help to manage the impact of new healthcare situations caused by possible supply shortages, new (and old) epidemics or possible pandemics.

Against the background of efficiency, it seems central to be able to reliably provide particularly the highly weighted indicators. Furthermore, it seems important to include the indicators that are important from a scientific point of view in the decision-making process through a multidisciplinary dialogue.

## Credit Author Statement

M.Sc. Nicolas Reinoso Schiller - Conceptualization, Methodology, Writing original draft, Formal analysis, Visualization, Resources.

M. Sc. Karina Usipbekova - Conceptualization, Writing original draft, Project administration, Resources.

Dr. Katja Hille - Conceptualization, Writing review and editing, Project administration, Methodology.

Dr. Johannes Dreesman - Conceptualization, Writing review and editing, Project administration.

M.Sc. Kill Schwarz - Conceptualization, Data curation, Formal analysis, Resources.

Dr. Karin Reimers - Conceptualization, Writing review and editing, Supervision.

Dr. Fabian Feil - Writing - Review &Editing, Supervision.

Prof. Dr. Simone Scheithauer - Conceptualization, Methodology, Writing - Review & Editing, Supervision.

## Footnotes

3 For its acronym in German: Niedersächsisches Landesgesundheitsamt (NLGA).

4 From the German: Universitätsmedizin Göttingen.

5 The German Interdisciplinary Association for Intensive Care and Emergency Medicine e. V. (with the German acronym DIVI) has been recording since april 2020 the free and occupied treatment capacities in intensive care medicine of around 1,300 acute hospitals in Germany on a daily basis.
