# Supplementary material for: Pandemic management: Analysis of availability and relevance of surveillance indicators by COVID-Task-Forces in the German federal state of Lower Saxony
Source: Infect Prev Pract. 2023 Jun 15;5(3):100294. doi: 10.1016/j.infpip.2023.100294 (PMC10485663; doi:10.1016/j.infpip.2023.100294)
Supplement: Multimedia component 1 [file mmc1.docx]

**List of Surveillance indicators**

**A) Hospital surveillance indicators:**

- Number of new patients admitted to hospital each day with suspected or confirmed SARS-CoV-2 infection.
- Total number of new patients admitted daily (independent of Covid-19)
- Number of people coming to the emergency department every day with suspected SARS-CoV-2 infection
- Number of people who are treated daily in the emergency department
- Number of occupied beds in Covid 19 wards
- Number of ICU beds occupied by Covid-19 patients
- Free capacities for further Covid 19 patients
- Number of free beds on intensive care units
- Number of beds on intensive care units
- Total number of respirators
- Number of occupied respirators
- Availability of care-related resources (such as rapid antigen tests, protective masks, etc.) in hospitals
- Proportion of staff absences in all Hospital departments due to illness or quarantine
- Proportion of absences among staff in emergency services due to illness or quarantine
- Proportion of absences among ambulance & rescue staff due to illness or quarantine

**B) Surveillance parameters relating to facilities for the elderly and nursing homes**

- Number of daily newly confirmed cases of SARS-CoV-2 infection among residents of inpatient geriatric and nursing facilities
- Number of daily newly confirmed cases of SARS-CoV-2 infections among employees of inpatient care and nursing facilities
- Number of current SARS-CoV-2-positive residents of inpatient geriatric and care facilities care
- Number of currently SARS-CoV-2-positive employees of in-patient care and nursing facilities for the elderly
- Number of deceased residents per time unit (for example, per day or per week)
